# Evaluation of Enamel Surface Roughness and Volumetric Change after Resin Remnant Removal following Orthodontic Bracket Debonding

**DOI:** 10.3290/j.ohpd.c_2117

**Published:** 2025-07-04

**Authors:** Bora Korkut, Kadir Emre Uzun, Cigdem Hacıali, Tuna Unal, Dilek Tagtekin

**Affiliations:** a Bora Korkut Associate Professor, Department of Restorative Dentistry, Marmara University, Istanbul, Türkiye. Conception, design, materials, data collection/processing, analysis, interpretation, literature review, writer.; b Kadir Emre Uzun Dentist, Private Practice, Istanbul, Türkiye. Literature review.; c Cigdem Hacıali Dentist, Department of Restorative Dentistry, Marmara University, Istanbul, Türkiye. Literature review, writer.; d Tuna Unal Assistant Professor, Department of Restorative Dentistry, Istanbul Health and Technology University, Istanbul, Türkiye. Design, literature review.; e Dilek Tagtekin Professor, Department of Restorative Dentistry, Marmara University, Istanbul, Türkiye. Conception, supervision, interpretation.

**Keywords:** debonding, dental microscope, iatrogenic damage, resin remnant, volumetric loss

## Abstract

**Purpose:**

To evaluate surface roughness and volumetric change of enamel after using different resin remnant removal (RRR) techniques, following orthodontic bracket debonding.

**Materials and Methods:**

Metal orthodontic brackets (Mini Twin Brackets, RMO) were bonded to 60 human (central or lateral) labial mid-third surfaces, and debonded 24 h after by a single orthodontist. The remaining composites were completely removed with the fluorescence light guidance by the D-Light-Pro led curing unit (GC/detection mode). The removal procedures were performed without magnification (n = 30) or with 20× magnification/5500 K illumination by a dental microscope (OMS2000, Zumax) (n = 30). Three RRR techniques were used: 12-bladed carbide bur (Horico), red-banded diamond bur (Horico), SofLex Disc (medium/40 μm, fine/24 μm, and superfine/8 µm; 3M). Surface changes were evaluated visually through microscope photographs by enamel surface index (ESI) and volumetrically by overlapping the three-dimensional images of a laser scanner device (LAS-20, SD-Mechatronik) in the Geomagic Design X (3D Systems) software. The deemed significance was set at <0.050 for the statistical analyses.

**Results:**

A positive, strong correlation was found between visual and volumetric change scores (P <0.001). Lesser volumetric loss (P <0.001) and roughness (P = 0.009) were observed for all RRR techniques when the magnification was used. Volumetric loss (mm^3^) by diamond bur was significantly the highest [1.85(1–3)a], followed by SofLex Disc [1.1(1–1)c] and carbide bur [0.59(0–1)b](P <0.001). Visual surface roughness scores (Ra) were statistically significantly higher for diamond bur [4.5(4–5)b](P <0.001), followed by carbide bur 2(1–3)a and SofLex Disc 1(1–2)a.

**Conclusion:**

Surface roughness should always be assessed together with the volumetric enamel loss for the selection of RRR technique. Red-banded diamond bur should not be used for RRR. Even though the least surface roughness can be provided by SofLex Disc system, it can provide more intact enamel surface loss than the carbide bur. Magnification was considered useful for the RRR to provide a smoother surface while better preserving the intact enamel tissue.

Chemical adhesion of brackets or attachments to enamel in orthodontic treatment is based on surface roughening with phosphoric acid, resulting in microporosity that allows micro-retention of the resin composite to infiltrate into the dental enamel.^1^ After the orthodontic treatment, these brackets or attachments are mechanically debonded. Regarding the debonding procedure, the separation can occur between the bracket base and the adhesive interface or the adhesive and the enamel interface. However, it can also occur within the adhesive.^25^ The mechanical removal of the remaining resin composite on the enamel surface is an important final step of the procedure, since the residual resin may accumulate dental plaque and thereby lead to discolouration and as a result, caries lesions.^13^

There are two main objectives clinically in the resin remnant removal (RRR) procedure, which are: to obtain a smooth/glossy enamel surface after the complete removal of the residual composite from the surface, and to avoid iatrogenic damage by preventing or minimising the loss of external enamel layer during the removal procedure.^3^ Previous studies focused on evaluating either the surface roughness,^8,36^ or the amount of remaining remnants.^9^ The polishing materials, as well as the number of polishing systems, varied in these studies. Moreover, different evaluation methods were used for the evaluations. However, a successful RRR procedure should remove the remnants completely and provide a smooth surface while preserving the intact enamel tissue underneath. Currently, there is no worldwide accepted clinical RRR technique to remove the composite remnants from the enamel surface without creating damage to the natural dental tissues.^19^ Due to the lower hardness of dental enamel than the abrasive surface finishing/polishing materials (aluminium oxide, zirconium oxide, quartz, carbon steel, diamond, and tungsten carbide) used on the enamel surface, either insufficient removal of the remnants or the iatrogenic surface damage may usually occur.^1^ The potential iatrogenic damages may cause postoperative sensitivity or unwilling aesthetic appearance, especially when observed on the vestibular surface of anterior teeth.^19^ Besides the type of abrasive surface finishing/polishing material, the use of magnification,^5,7,37^ as well as the clinician’s experience,^19^ are considered the influencing factors on the polishing quality and the iatrogenic damage.^1,3^

The clinical diagnosis of the remaining composite on the enamel surface can be difficult due to the high colour adjustment potential of recent resin composite materials.^3^ The use of coloured adhesive resin composites or fluorescence detection light units may be useful during the removal procedure.^3,19^ Moreover, it is very difficult to assess the roughness of the enamel tissue clinically.^19^ However, working under proper magnification/illumination or with the guidance of dental photography techniques may overcome this problem.^7^

Clinicians may select a wide range of RRR techniques clinically; however, fine-grit diamond burs,^40,41^ tungsten-carbide burs,^3,16,37,38,40^ and composite aluminium-oxide polishing discs^14,25,39,40^ are the most commonly used materials to remove residues from the surface. This *in-vitro* study aimed to evaluate the surface roughness and volumetric change of dental enamel tissue after the complete removal of the resin remnants by these three RRR techniques, following the orthodontic bracket debonding procedure. Also, it aimed to investigate the effect of magnification on the RRR procedure. The null hypotheses of the study were: (1) the surface roughness is not affected by the RRR technique; (2) the intact enamel surface is not affected by the RRR technique; (3) the use of magnification does not affect the level of surface roughness and volumetric loss.

## MATERIALS AND METHODS

**Fig 1:**
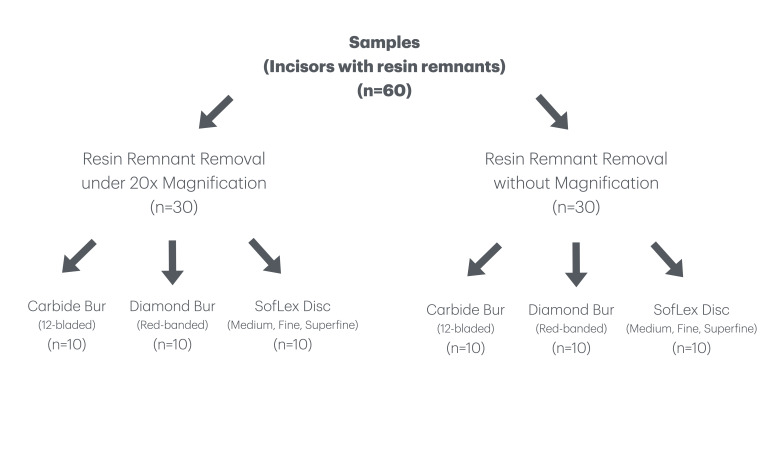
Flowchart of the study.

This study was approved by a local ethical committee (no: 09.2023.1347 and date: 14.11.2023). The flowchart of the study is presented in Figure 1.

### Preparation of the Samples

A minimum of 60 samples was required for this study for a power of 80% (1-β) and a confidence interval of 95% (1-α), with an effect size of w = 0.356 and a significance level of 0.05 (G*Power V.3.1.9.6, Germany). Accordingly, 60 extracted human maxillary central incisors (at an average age of 56) were collected from a university clinic. The reason for the extractions was due to periodontal disease. The teeth in any colour with intact crowns were included in the study. The ones including composite restorations, caries, hypomineralisation, erosion, and fluorosis lesions were excluded from the study. Then, selected tooth surfaces were cleaned with the prophylaxis paste, and all the teeth were kept in 0.1% thymol solution for disinfection. Standardised acrylic cylindrical blocks of 5 cm in diameter and 3 cm in height were prepared using custom metal moulds to fix the samples. Then the teeth were placed horizontally in the blocks, leaving the labial surfaces on the top with no acrylic covering (Fig 2). After storing the samples in water at room temperature for a day, 500 cycles of thermal cycling from 5 to 55 °C were performed, with a dwell time of 20 s. The transfer time between the baths was 5–10 s.

Before the orthodontic bracket placement, all samples were scanned by using a laboratory laser surface scanner device (LAS-20, SD-Mechatronik, Münich, Germany) with 0.01 mm sensitivity, generating initial three-dimensional volumetric data for each sample. The samples were placed in the scanner one by one, and the surfaces were scanned by the device before the RRR procedures. Also, the initial 20×-magnified dental microscope photographs of each sample were taken at 5500 K illumination. A direct and an inclined photograph was recorded for each sample.

Following that, the labial mid-third surfaces of the samples were roughened with 37% phosphoric acid (OpalEtch, Ultradent Products, ABD) for 30 s, rinsed, and dried. The Transbond XT Light-cure Adhesive Primer (3M) was applied to the roughened surfaces by rubbing for 10 s, refined with air pressure to create a thin layer, and polymerised for 10 s. A LED curing unit (Valo Grand, Ultradent Products) was used with irradiation of 1600 mW/cm^2^ for polymerisation. Metal orthodontic brackets (0.022-inch Mini Twin Brackets, Rocky Mountain Orthodontics [RMO], France) for maxillary and mandibular central incisors were bonded on the labial mid-third surfaces of the teeth by using the Transbond XT Light-cure Adhesive Paste (3M). The adhesive amounts were standardised by applying them to the enamel using a silicone mould (Mini-Mold small wire bonder, G&H Orthodontics, USA).^4^ The excessive adhesive composite material surrounding the brackets was removed gently by using a dental instrument. Finally, all samples (n = 60) were polymerised for 10 s at each of the mesial and distal sides (Fig 3). All the procedures were performed by a single orthodontist. The samples were kept in distilled water for 24 h.

### Experimental Groups and RRR Techniques

All the brackets were de-bonded by the orthodontist after 24 h using a Howe Plier (Hu-Friedy Group, USA) (Fig 4). The resin remnants on the sample surfaces following the debonding procedures were evaluated using the Adhesive Remnant Index (ARI) in the present study to ensure standardisation before the cleaning procedures.^15,29^ All the samples received an ARI score of 3 (10–90% of adhesive remaining). Then the resin remnants were removed by a single experienced restorative dentistry instructor, with the guidance of fluorescence-aided identification technique (FIT) by using the D-Light Pro (GC, Japan) device at 400 nm wavelength to ensure the residues were removed from the surface completely.^24^ This device can indicate the remaining resin composites on the enamel surface clinically by fluorescence illumination ability; therefore, the clinician is always sure to completely remove the composite residues from the surface.

The resin remnants on 30 teeth were removed either without magnification or the resin remnants on another 30 teeth were removed by using a dental microscope (Zumax OMS2000, China) with a 20× magnification/5500K illumination (n = 30). The three different RRR techniques were used as subgroups for all the teeth to remove the composite residues completely: (1) a multi-blade (12 blades) tungsten-carbide bur (C48L/314, Horico, Germany); (2) a red-banded diamond bur (FG/199C, Horico); (3) SofLex Disc (medium/40 μm, fine/24 μm, and superfine/8 µm, respectively, 3M) (n = 10 for each group) (Fig 5 and Fig 6).

All the RRR procedures were performed by using a low-speed electric motor with a handpiece at 5000 rpm without water cooling. Water cooling was not used during the low-speed polishing procedures to see the specific surface roughness changes precisely, as suggested in previous studies.^19,20^ Finer grits of SofLex Disc were used for surface subtraction while working closer to the enamel tissue. All the RRR materials (burs and discs) used were renewed for each sample.

### Assessment of Enamel Surface

Surface structures were assessed visually and volumetrically after the surface RRR techniques. A pilot study was performed by two operators with 24 samples other than the research samples for the visual and volumetric assessments. According to Cohen’s kappa statistics, a good positive inter-observer agreement (ICC kappa value of 83 and 87 for visual and volumetric assessments, respectively) was found between the two operators. The intra-observer agreements for the first and second operators were also good (ICC kappa values of 92/94 and 93/96 for visual and volumetric assessments, respectively), thereby all the assessments of the main research were performed by the first operator.

**Fig 7:**
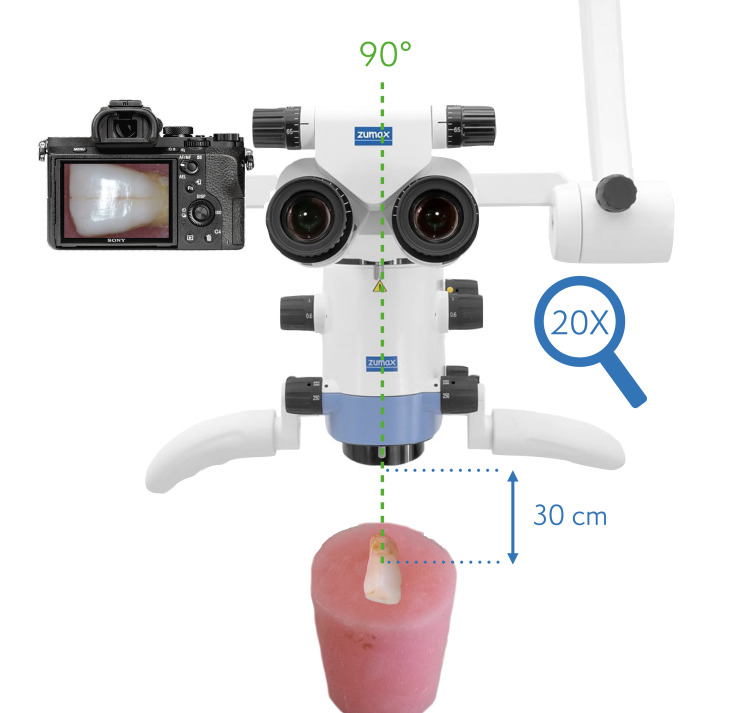
Dental operating microscope setup (vertical positioning) for direct, frontal images.

Regarding the visual assessments, the operator analysed the enamel surface structures through the 20×-magnified dental microscope (Zumax OMS2000, China) images. The microscope was used with integrated microscope illumination (Continuous TrueTone LED, Zumax) in full-power and at 5500°K temperature. Images were taken using an integrated full-frame and mirrorless camera (Alpha 7iii, Sony, Japan) with constant settings (ISO 100, F24, shutter speed 1/250, and WB 5500°K). Direct labial surface images (frontal images) of each tooth were taken by positioning the microscope vertically to the tooth surface (Fig 7).

**Fig 8:**
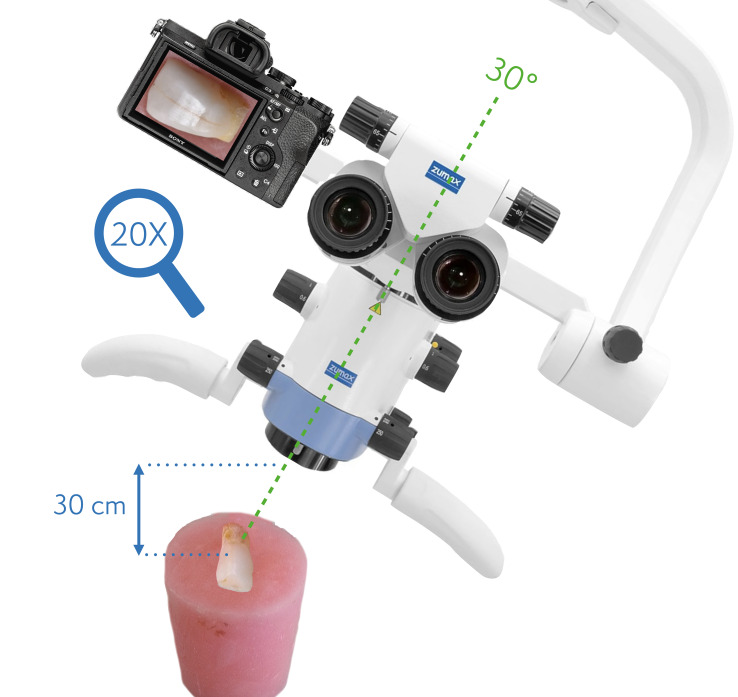
Dental operating microscope setup (30º inclined positioning) for inclined images.

In addition to the direct labial surface images, inclined photographs were taken by inclining the microscope 30º to the tooth surfaces (Fig 8). The flat acrylic cylindrical blocks’ base was fixed on a flat table, and both the vertical and inclined images were recorded from a 30 cm distance to the tooth surface using the same camera and illumination setups.

A surface roughness grading system, the enamel surface index (ESI) by Zachrisson and Arthun, was used to generate visual scores through the images (Table 1).^3,16,26,42^ This system was a pioneer of many further grading systems, and similar modified systems were later used by Howell and Weekes,^11^ Hong and Lew,^10^ and Schuler and van Waes.^31^

**Table 1:** Enamel surface roughness grading system for visual scoring

Enamel surface Index (ESI)^3,16,26,42^
**Grade 0**	Perfect surface with no scratches and distinct intact perikymata.
**Grade 1**	Satisfactory surface with fine scratches and some perikymata.
**Grade 2**	Acceptable surface several marked and some deeper scratches, no perikymata.
**Grade 3**	Imperfect surface several distinct deep and coarse scratches, no perikymata.
**Grade 4**	Unacceptable surface with coarse and deeply marked appearance.


The volumetric analyses were performed by the same operator using the dental laboratory laser scanner device. Each sample was placed in the scanner, and the surfaces were scanned by the device after the RRR procedures. The collected data of each sample were uploaded into a compatible computer software, Geomagic Design X (3D Systems, USA). The initial and final volumetric data were overlapped automatically by the software, and the volumetric differences were expressed in mm^3^ (Fig 9).

Statistical analyses were performed with the IBM SPSS V23 software program. The normality was analysed by the Shapiro–Wilk test. The analyses of the data were performed by using Spearman’s Rho Correlation Coefficient, Kruskal–Wallis H test, and Mann–Whitney U test. The deemed significance was set at <0.050.

## RESULTS

The RRR technique, the use of magnification, and their interactions were considered effective factors in volumetric change (P <0.001 for each).

Regardless of the magnification, the red-banded diamond bur group presented statistically significantly higher [1.85 (1–3)a] volumetric change than the SofLex Disc group [1.1 (1–1)c], which was also significantly higher than the tungsten-carbide bur [0.59 (0–1)b] (Table 2). With and without the use of magnification, the obtained volumetric changes of the red-banded diamond bur were also statistically significantly higher than the SofLex Disc (P <0.001 for each), which was followed by the tungsten-carbide bur (P <0.001 for each). The use of magnification statistically significantly decreased the volumetric change for all three RRR techniques (P <0.05 for each) (Table 2). The obtained volumetric changes by red-banded diamond bur without magnification were significantly the highest [2.36 (2–3)A] among all, while the changes were statistically significantly the lowest [0.35 (0–0)D] for the tungsten-carbide bur group under magnification.

**Table 2:** Comparisons of RRR techniques with and without magnification in terms of the volumetric changes (mm^3^)

Magnification	RRR techniques	Total	P*
Red-banded diamond	Tungsten- carbide	SofLex disc
No	2.36 (2–3)^A^	0.9 (1–1)^C^	1.19 (1–1)^E^	1.19 (1–3)	**<0.001**
Yes	1.54 (1–2)^B^	0.35 (0–0)^D^	1 (1–1)^C^	1 (0–2)	**<0.001**
Total	1.85 (1–3)^a^	0.59 (0–1)^b^	1.1 (1–1)^c^	1.1 (0–3)	**<0.001**
A to E: No significant differences among the interactions with same letter; a to c: No significant differences between the resin remnant removal (RRR) techniques with the same letter. Median (min. – max.)

Regardless of magnification, the red-banded diamond bur presented statistically significantly higher [3.5 (3–4)b] ESI scores than the tungsten-carbide bur [1 (0–2)a], which were similar to the SofLex Disc’s [0 (0–1)a] (Table 3). With and without the use of magnification, the visual scores of the red-banded diamond bur group were statistically significantly higher than the scores of the tungsten-carbide bur group (P <0.001 for each), which were similar to the scores of the SofLex Disc group (P ≥0.05 for each). The use of magnification statistically significantly decreased the visual scores for the red-banded diamond bur, the tungsten-carbide bur, and the SofLex Disc (P <0.001, P = 0.001, P = 0.023, respectively) (Table 3). The scores of red-banded diamond bur without magnification were statistically significantly the highest [4 (4–4)b] among all, while the scores were statistically significantly the lowest [0 (0–0)a] for the SofLex Disc under magnification.

**Table 3:** Comparisons of resin remnant removal techniques with and without magnification in terms of the ESI scores

Magnification	Red-banded diamond	Tungsten- carbide	SofLex disc	Total	Test stat.	P*
No	4 (4 – 4)b	2 (1 – 2)a	1 (0 – 1)a	2 (0 – 4)	25.700	**<0.001**
Yes	3 (3 – 3)b	1 (0 – 1)a	0 (0 – 0)a	1 (0 – 3)	25.375	**<0.001**
Total	3.5 (3 – 4)b	1 (0 – 2)a	0 (0 – 1)a	1 (0 – 4)	46.625	**<0.001**
Test stat.	0.000	9.000	20.000	279		
P**	**<0.001**	**0.001**	**0.023**	**0.009**		
*Kruskal–Wallis H test, **Mann–Whitney U test, a, b: No significant difference between the resin remnant removal (RRR) techniques with the same letter.

Regarding the correlation between the obtained volumetric changes and the enamel surface index scores in terms of the RRR techniques, a fairly strong positive relationship was found for the tungsten-carbide and red-banded diamond burs (P <0.001 for each), and a moderate positive relationship was found for the SofLex Disc (P = 0.007) (Table 4).

**Table 4:** Correlation between the volumetric changes and ESI scores, in terms of the RRR techniques

	r	P
Red-banded diamond	0.867	**<0.001**
Tungsten carbide	0.927	**<0.001**
SofLex disc	0.587	**0.007**
**Total**	0.708	**<0.001**
r: Spearman’s rho correlation coefficient.

### DISCUSSION

All three hypotheses of the study were rejected. The level of surface roughness and intact enamel loss varied among the investigated RRR techniques. Additionally, both were affected by the use of microscope magnification. This study revealed that the RRR materials should be evaluated by both the effectivity in surface smoothening and enamel surface preservation.

Following the orthodontic bracket debonding, there is no consensus on a non-invasive RRR technique yet to remove the composite remnants completely from the enamel surface, clinically.^24,32^ The majority of the previous studies focused on the surface roughness and/or damage of the enamel surface, but there is only limited scientific evidence on investigating the amount of dental tissue loss from the surface following the finishing and polishing procedures.^12,13,19,31,33,42^ Even when a smooth enamel surface is generated, the enamel thickness might be decreased due to the use of rotary instruments.^11^ Iatrogenic external enamel surface removal may lead to a decrease in enamel resistance to demineralisation as a result of exposing enamel prism endings.^12^ Whereas, resin infiltration resulting from the etching of the enamel during the orthodontic bracket placement might be up to 50 µm.^4^ Thus, from a point of view, a slight enamel surface removal might be considered clinically tolerable to achieve complete adhesive removal. The rotary instruments used for the removal may cause enamel abrasion depending on the composition and size of the rotational speed, abrasive particles, and the press-on force.^12,18^ The ideal clinical technique must provide complete adhesive removal to obtain the best surface smoothness while preserving the surface enamel thickness at most.^24^

Various RRR techniques were mentioned as a clinical option previously.^1,16,24^ A fine (red-banded) diamond bur was considered the most commonly used (3.8% among all) diamond bur among orthodontists for RRR, and therefore it was selected for the study.^41^ Also, the aluminium-oxide-coated SofLex Disc system, except the coarse-grit disc, was selected as a gold standard for the restorative surface finishing and polishing procedure.^19,20^ Regarding the multibladed burs, there is evidence claiming no difference among the different blade numbers.^16,41^ Webb et al considered the 12-bladed carbide bur the most commonly used (49.3% among all) bur among orthodontists for RRR, and no difference in 12- and 20-bladed carbide burs.^41^ Ferreira et al supported that by presenting no difference among 6-, 12-, and 30-bladed burs, but also mentioned that the 6-bladed had a more unsatisfactory performance for the preservation of enamel tissue.^16^ Therefore, a 12-bladed carbide bur was selected in this study as one of the RRR techniques. All the RRR techniques were also used in dry conditions to simulate the reported common way of clinical use.^39^ In addition, the guidance of FIT was previously considered superior in the clean-up procedure after orthodontic debonding regarding the time needed and the effectiveness.^24,30,34,35^ Auxiliary devices such as FIT were determined to be effective for the RRR in terms of preserving the healthy tooth structure.^24^ Thus, the resin remnants were removed completely by the guidance of FIT to eliminate the parameter of the remaining composite for the assessments.

The enamel loss following the debonding procedure might occur due to the bonding failure between the bracket’s surface and the adhesive, within the adhesive composite, or between the enamel and the adhesive composite.^24^ There are different results in the previous *in-vitro* studies regarding the amount of post-cleaning enamel tissue loss.^12^ Enamel loss depth ranging from 29.5 to 41.2 µm after RRR was reported by Pus and Way.^27^ 50 µm mean value was reported by Al Shamsi et al,^2^ while only 5 to 10 µm was reported by Zacharison and Arthun.^42^ In this study, the selected RRR technique affected the level of surface roughness and volumetric loss, consistent with many previous studies,^18,39,41^ considering the first hypothesis rejected. Regarding the enamel surface index scores, the red-banded diamond bur statistically significantly provided both the highest roughness on the enamel surface (Table 3) and the highest volumetric loss from the enamel surface (Table 2). These results supported the findings of a previous systematic review, which concluded that diamond burs should not be used for adhesive removal.^12^ Although scoring visually by the enamel surface index is subjective, interpreting the enamel surface through light reflections on a 20× magnified, sharp, and clear dental microscope image might be more informative compared to contact profilometry or laser scanners. A systematic review by Janiszewska-Olszowska et al mentioned that contact profilometry has an important limitation due to the stylus, and the laser scanner can’t be used to scan shiny surfaces.^12^ They also mentioned the lack of volumetric analysis regarding this topic and suggested to use of volumetric quantitative evaluation to assess the amount of enamel loss after RRR techniques.

Although the SofLex Discs were determined to provide the smoothest surface for resin composite polishing *in-vitro* studies,^17,20^ multi-blade tungsten-carbide burs were also considered an effective and safe method for RRR when used carefully at low speed.^1,13,14^ Consistent with that, in the present study, tungsten-carbide bur (12 blades) and SofLex Disc (medium, fine, superfine grits) provided statistically similar surface roughness, and both were better than the red-banded diamond bur (Table 3). Although the SofLex Disc provided slightly smoother surfaces, supporting the results of Roush et al^28^ and Albertini et al^1^ the differences in surface roughness were not significant compared to the carbide bur, consistent with the results of Melvin et al.^21^ Although there are some previous findings mentioning tungsten-carbide burs to cause greater enamel surface damage than the SofLex Disc, like the *in-vitro* study of Cesur et al^6^ type and surface convexity of the teeth might have influenced the result, as they used only the premolar teeth for the investigation.

The volumetric loss of enamel after debonding ranged between 0.02 ± 0.01 mm^3^ and 0.61 ± 0.51 mm^3^ among previous studies.^6,34^ Tüfekçi et al reported a greater loss of enamel for the SofLex Discs (medium and fine grits; 0.14 mm^3^) than slow-speed tungsten-carbide burs (0.11 mm^3^).^38^ Our volumetric laser scanner assessments also supported these previous findings, while greater losses were detected in the present study. The greater losses in the present study might be related to the complete removal of the resin remnants from the surface by the guidance of FIT, instead of unaware leaving and polishing some on the surface. The highest volumetric loss of enamel tissue was 1.85 mm^3^ for the red-band diamond bur. It was 1.1 mm^3^ for the SofLex Disc, which was significantly greater than the tungsten-carbide bur of 0.59 mm^3^ (Table 2). Therefore, the previous statement of Ulusoy was supported by the result that, following the use of even tungsten-carbide burs or multi-step discs for RRR, enamel scratching is inevitable.^39^ The three-step SofLex Disc system was considered quantitatively the most effective regarding surface smoothening, whereas the 12-bladed slow-speed tungsten-carbide bur was considered the safest regarding healthy enamel tissue preservation. Thus, the second hypothesis was rejected. Our results strongly agree with Koh et al,^18^ who reported that surface roughness is determined by the characteristics of the RRR technique.

The use of magnification enhanced the effectiveness of all three investigated RRR techniques on both surface smoothening and preservation of the enamel tissue, consistent with several previous studies,^1,5,7,22^ which resulted in the rejection of the third hypothesis of the study. This result is consistent with many previous reports, such as Montasser and Drummon^23^ mentioning that the dental microscope magnification of 20× provided a more accurate evaluation for RRR, Thawaba et al^37^ reporting the use of loupe magnification enhanced the clean-up procedure by reducing enamel surface roughness, and Ghaleb et al^9^ considering the loupe magnification an effective tool for RRR with less surface roughness and enamel damage.

It might be interpreted that the level of surface smoothing and surface preservation may not be directly proportional every time. The correlation results among the RRR techniques supported this unusual idea (Table 4). The results revealed that surface roughness and volumetric loss were directly proportional for one-step diamond bur and carbide bur, but not for the three-step SofLex Disc, even without using the coarse-grit disc. Probably the medium-grit disc created a rougher enamel surface first when removing the resin remnants, and then fine-/ultrafine-grit discs re-smoothened the surface gradually by removing more intact tissue. Although total working time and the operator-related factors might also have an effect on this, the damage of an RRR technique can’t be assessed by investigating only the surface roughness. ‘First, do no harm’ should always be the main objective of a dentist. Therefore, together with the surface roughness, the amount of irreversible volumetric enamel loss should be considered when comparing the clinical effect and quality of finishing and polishing systems.

This *in-vitro* study has some limitations, mainly due to not perfectly simulating the clinical intraoral conditions such as saliva, oral hygiene, temperature, and pH, which can all have an impact on the results. The variety of RRR techniques might be extended, and their combinations might be investigated. The removal of the composite residues is an operator-dependent procedure due to the level of experience and the press-on force variable.^12,20,41^ It might be beneficial to also evaluate the surface roughness by using a contact profilometer. It might be more accurate to standardise the press-on force during the removal procedure for further research. The combinations of different RRR techniques may result in different outcomes. Additionally, the type of tooth and the related orthodontic bracket may affect the outcome due to the different convexity on the labial surface.^6,12,24^ Thus, it might be better to include canines, premolars, and molars for further research.

## CONCLUSION

Within the limitations of this study, the red-band diamond burs should not be used for the RRR. A smooth enamel surface can be provided by using either the three-step SofLex Disc or a 12-bladed tungsten-carbide bur, whereas the SofLex Disc may provide a slightly smoother surface. However, the SofLex Disc also causes a greater intact enamel tissue loss than the 12-bladed carbide bur during the RRR procedure. Therefore, the clinician should always assess the surface roughness together with the volumetric loss for the selection of the proper RRR technique. Microscope magnification can be recommended during the RRR procedure as it can aid in providing better surface smoothening and preservation of intact enamel tissue.

### Statements and Declarations

**Funding:** None.

**Competing interests:** The authors have no financial or non-financial interests that are directly or indirectly related to the work submitted for publication.

#### Consent for publication

Not applicable.

#### Ethics approval

This *in-vitro* study was approved by Ethics Committee of Marmara University Institute of Health Sciences (protocol number 09.2023.1347 and date 14.11.2023).

#### Data availability

The data and materials of the study are available from www.eistatistik.com.

**Fig 3a to f:** Images of the samples with bonded orthodontic metal brackets.

**Fig 2a to f:** Initial images of the samples at 20× magnification and 5500 K° illumination.

**Fig 6a to f:** Inclined images for enamel surface characteristics after completing RRR. (a) RRR with tungsten-carbide bur without magnification; (b) RRR with red-banded diamond bur without magnification; (c) RRR with SofLex Disc without magnification; (d) RRR with tungsten-carbide bur under 20× magnification; (e) RRR with red-banded diamond bur under 20× magnification; (f) RRR with SofLex Disc under 20× magnification.

**Fig 5a to f:** Enamel surface characteristics after completing the RRR procedure. (a) RRR with carbide bur without magnification; (b) RRR with red-banded diamond bur without magnification; (c) RRR with SofLex Disc without magnification; (d) RRR with carbide bur under 20× magnification; (e) RRR with red-banded diamond bur under 20× magnification; (f) RRR with SofLex Disc under 20× magnification.

**Fig 4a to f:** Images of the samples with resin remnants on the enamel tissue after de-bonding of the brackets.

**Fig 9a to c:** Geomagic Design X images of a sample surface. (a) 3D surface topography; (b) overlapped initial and final volumetric data; (c) 3D image after trimming the edges for quantitative calculation.












